# A digoxin derivative that potently reduces intraocular pressure: efficacy and mechanism of action in different animal models

**DOI:** 10.1152/ajpcell.00617.2023

**Published:** 2024-04-01

**Authors:** Veluchamy Amutha Barathi, Adriana Katz, Shashikant Chaudhary, Hoi-Lam Li, Daniel M. Tal, Arie Marcovich, Chi-Wai Do, Steven J. D. Karlish

**Affiliations:** ^1^Translational Pre-Clinical Model Platform, Singapore Institute of Eye Research (SERI); ^2^ACP in Ophthalmology & Visual Sciences, DUKE-NUS Graduate Medical School, Singapore; ^3^Department of Ophthalmology, Yong Loo Lin School of Medicine, National University of Singapore, Singapore; ^4^Department of Biomolecular Sciences, Weizmann Institute of Science, Rehovot, Israel; ^5^School of Optometry, The Hong Kong Polytechnic University, Hong Kong, People’s Republic of China; ^6^Opthalmology Department, Kaplan Medical Center, Rehovot, Israel; ^7^Hebrew University Medical School, Jerusalem, Israel; ^8^Centre for Eye and Vision Research (CEVR), Hong Kong, People’s Republic of China; ^9^Research Institute for Smart Ageing (RISA), The Hong Kong Polytechnic University, Hong Kong, People’s Republic of China

**Keywords:** digoxin derivative, intraocular pressure, Na,K-ATPase

## Abstract

Glaucoma is a blinding disease. Reduction of intraocular pressure (IOP) is the mainstay of treatment, but current drugs show side effects or become progressively ineffective, highlighting the need for novel compounds. We have synthesized a family of perhydro-1,4-oxazepine derivatives of digoxin, the selective inhibitor of Na,K-ATPase. The cyclobutyl derivative (DcB) displays strong selectivity for the human α2 isoform and potently reduces IOP in rabbits. These observations appeared consistent with a hypothesis that in ciliary epithelium DcB inhibits the α2 isoform of Na,K-ATPase, which is expressed strongly in nonpigmented cells, reducing aqueous humor (AH) inflow. This paper extends assessment of efficacy and mechanism of action of DcB using an ocular hypertensive nonhuman primate model (OHT-NHP) (*Macaca fascicularis*). In OHT-NHP, DcB potently lowers IOP, in both acute (24 h) and extended (7–10 days) settings, accompanied by increased aqueous humor flow rate (AFR). By contrast, ocular normotensive animals (ONT-NHP) are poorly responsive to DcB, if at all. The mechanism of action of DcB has been analyzed using isolated porcine ciliary epithelium and perfused enucleated eyes to study AH inflow and AH outflow facility, respectively. *1*) DcB significantly stimulates AH inflow although prior addition of 8-Br-cAMP, which raises AH inflow, precludes additional effects of DcB. *2*) DcB significantly increases AH outflow facility via the trabecular meshwork (TM). Taken together, the data indicate that the original hypothesis on the mechanism of action must be revised. In the OHT-NHP, and presumably other species, DcB lowers IOP by increasing AH outflow facility rather than by decreasing AH inflow.

**NEW & NOTEWORTHY** When applied topically, a cyclobutyl derivative of digoxin (DcB) potently reduces intraocular pressure in an ocular hypertensive nonhuman primate model (*Macaca fascicularis*), associated with increased aqueous humor (AH) flow rate (AFR). The mechanism of action of DcB involves increased AH outflow facility as detected in enucleated perfused porcine eyes and, in parallel, increased (AH) inflow as detected in isolated porcine ciliary epithelium. DcB might have potential as a drug for the treatment of open-angle human glaucoma.

## Graphical abstract

**Figure:**
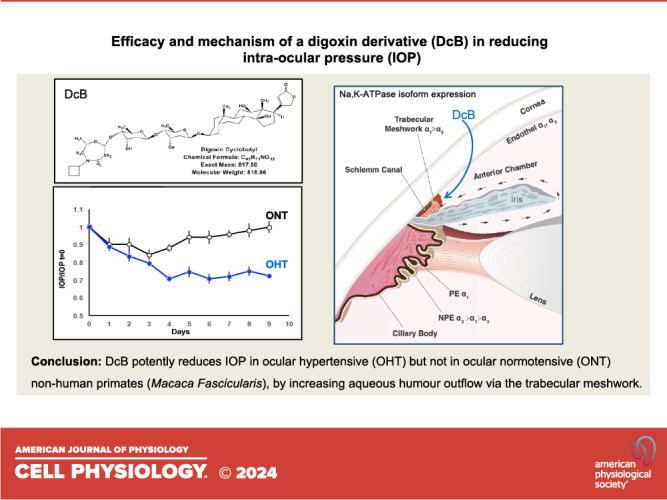

## INTRODUCTION

Glaucoma is a heterogeneous group of ocular disorders characterized by progressive optic nerve damage and visual field loss and is a significant cause of irreversible blindness. Raised intraocular pressure (IOP) is the major modifiable risk factor for glaucoma and is associated with glaucoma development and disease progression (1–4). The primary focus of drug treatment is reduction of intraocular pressure, avoidance of surgical intervention, and minimization of the risk of damage to the optic nerve and blindness.

Current glaucoma medications include several classes of topical drugs that reduce IOP by distinct mechanisms (5, 6). For example, prostaglandin analogs (e.g., latanoprost) enhance uveoscleral aqueous humor (AH) outflow, β-blockers (e.g., timolol), α agonists (e.g., brimonidine) and carbonic anhydrase inhibitors (e.g., dorzolamide) decrease AH inflow, and Rho kinase inhibitors (e.g., netarsudil) facilitate trabecular meshwork AH outflow. Despite these options, there is a need for new glaucoma drugs, due to loss of control of IOP over time and side effects of currently used drugs, exacerbated by patient noncompliance. Novel drugs may offer neuroprotection, longer durations of action, reduced side effects, and potential for combination therapies.

We have developed a family of perhydro-1,4-oxazepine derivative of the cardiac glycosides digoxin and digitoxin, the selective inhibitors of Na,K-ATPase, used classically to treat heart failure, and assessed their possible use as topical agents for reducing intraocular pressure (7–10). The derivatives were developed on the basis of a screen of purified human Na,K-ATPase isoforms (α1β1, α2β1−3, α3β1) with various cardiac glycosides from which it was concluded that modification of the digitoxose residues could lead to isoform-selective inhibitors (10). Indeed, several of the perhydro-1,4-oxazepine derivatives of digoxin showed distinct selectivity for all α2β1−3 complexes isoforms over α1β1, especially the cyclobutyl derivative of digoxin, DcB (8, 9). Supplemental Figure S1 shows the structure of DcB compared with digoxin (11). In addition to its strong selectivity for the α2 isoform of human Na,K-ATPase, DcB is also much more lipid soluble than digoxin (clogP 2.93 compared with 1.42, respectively).

The hypothesis underlying this project can be summarized as follows. It has been shown that in functional syncytium of ciliary epithelium (12) the nonpigmented (NPE) cells express mainly α2β2 or α2β3 isoform complexes of Na,K-ATPase, with only minor amounts of α1β1, whereas the pigmented epithelium (PE) cells express mainly α1β1 (8, 13, 14) (see also Supplemental Figs. S3 and S4). Other tissues in the anterior chamber of the eye, which could come into contact with a topically applied digoxin derivative, for example, cornea or lens express α1 and α3 but no detectable α2 (15, 16). Thus, it was hypothesized that a topically applied α2-selective cardiac glycoside, able to permeate the cornea, would inhibit the α2β2 or α2β3 pumps in NPE cells that power the transepithelial ion and fluid transport, and so reduce AH inflow and IOP, but would not significantly affect other tissues of the eye (7, 8).

The hypothesis just presented has been tested in New Zealand rabbits by looking at the effects on IOP of various perhydro-1,4-oxazepine derivatives of digoxin, compared with digoxin itself, or other cardiac glycosides such as ouabain. The experiments showed that both basal IOP (14–15 mmHg) and pharmacologically raised IOP (22–24 mmHg), induced by prior application of 4-amino pyridine, 4-AP (7, 8, 17),^1^ are effectively lowered by the topically applied digoxin derivatives, with an efficacy that increased with the degree of α2-selectivity. The cyclobutyl derivative, DcB (see Supplemental Fig. S1), which has the highest selectivity ratio of 20–30-fold for α2 over α1 isoforms, was the most efficacious of all derivatives tested in terms of the lowest effective dose and also the extent and duration of the effect (8). By comparison digoxin itself and ouabain were much less effective in all conditions and, unlike DcB, had little or no effect on basal IOP (7). The findings appeared to confirm an important role of the α2 isoform in AH formation and the proposed mechanism of action of DcB or other derivatives. Nevertheless, despite these tentative conclusions, it could not be excluded that the drug reduces IOP by increasing the AH outflow. This caveat arises because the drug’s efficacy in vivo should depend on more than one property, including both binding affinity to the drug target and permeability through the cornea (as well, possibly, as other pharmacokinetic factors), and it is difficult to distinguish between these factors in the in vivo setting. Indeed, the most α2-selective derivative (DcB) was also predicted to be highly lipid-soluble (clogP 2.93) and presumably, therefore, permeable through the cornea, whereas digoxin (clogP 1.42) is less lipid-soluble and should not be very permeable through the cornea. In addition, cardiac glycosides such as ouabain and strophanthidin have been shown to increase AH outflow in porcine tissue and experimental cellular models (18, 19).

The current work extends our previous findings and addresses the question of efficacy and mechanism of action of DcB in different animal models. Although the experiments with rabbits provided convincing evidence for the efficacy of DcB, it was desirable to test the compound on another animal model, particularly as there is evidence that the mechanism of AH secretion is not identical in all animals (20, 21). Thus, the choice fell on the nonhuman primate (NHP) species *Macaca fascicularis*. Importantly, a small but significant percentage of the *M. fascicularis* colony at the Singapore Eye Research Institute (∼5%) has been found to display spontaneous ocular hypertension (IOP 23–27 mmHg, the OHT-NHP model) compared with the normotensive population (IOP 14–16 mmHg, the ONT-NHP model) and a reduced aqueous humor flow rate (see also Table 1), while other clinical features are similar in the OHT-NHP and ONT-NHP. The basal IOP in the macaque monkeys is similar to that in humans (22), as is the range in ocular hypertension, and spontaneous ocular hypertension is a characteristic feature of most forms of human glaucoma (normal tension glaucoma excepted). Thus, the OHT-NHP may represent an optimal animal model for testing a drug such as DcB, with the potential for treatment of human glaucoma (23, 24), and for comparison of effects with those on the ONT-NHP. As a prior example of the utility of the OHT-NHP model in drug testing, these animals were used previously to characterize and compare the effects of Xalatan drops on IOP, with a sustained release formulation of the drug (24).

We have also made use of the New Zealand rabbit model (7, 8) to compare the effects of DcB on IOP with those of commonly used topical drugs, especially with the first-choice drug, latanoprost, when used alone or combined. These experiments also provide some insights into the mechanism of action of DcB.

To characterize the mechanism of action of DcB in more detail, studies of the separate effects of DcB on AH inflow and outflow pathways are necessary and have been carried out with a cellular preparation of porcine ciliary body for the assessment of AH secretion and electrical parameters, and direct measurement of outflow facility in ex vivo porcine eyes. These preparations simplify the analysis of the mechanism of action of DcB by having direct access to the target sites and avoiding corneal permeation (25, 26). The experiments have been designed to examine directly whether DcB exerts its IOP-lowering effect either by reducing AH secretion, enhancing outflow facility, or by both mechanisms.

Although DcB lowers IOP effectively in both rabbits and OHT-NHP in vivo, it now appears that the mechanism of action proposed previously (7, 8) must be revised. The data presented here points to raised AH outflow facility rather than decreased AH inflow as the mechanism of DcB-mediated lowering of IOP.

## MATERIALS AND METHODS

### Materials

8-Br-cAMP, ouabain, and heptanol were purchased from Sigma-Aldrich. Digoxin (D6003) and 4AP (A78403) were obtained from Sigma-Tau, Latanoprost (XalatanTM, from Pfizer), and other drugs (timolol and dorzolamide) from clinical laboratories.

### Drug Preparation

DcB and other perhydro-1,4-oxazepine derivatives of digoxin and digitoxin were synthesized, purified, and crystalized as described in previous publications (8, 9, 11). Before use DcB was dissolved in a standard polysorbate vehicle consisting of: 0.1% Tween 80; 0.3% (12 mM) Na-Citrate, pH-6.8; 0.8% (137 mM) NaCl (referred to as *vehicle A*) or polysorbate vehicles consisting of 0.1% Tween 80; 0.3% (12 mM) Na-Citrate, pH-6.8; 230 mM NaCl, or 100 mM NaCl plus 130 mM KC l, or 230 mM NaCl plus 100 mM KCl, or 290 mM KCl, as indicated in Fig. 4. The vehicle with 100 mM NaCl and 130 mM KCl was used for many experiments and in figure legends it is referred to as *vehicle B*. Other compounds and drugs were dissolved in distilled water before use.

### Nonhuman Primate Models

#### Animals.

Seven ocular hypertensive monkeys (OHT-NHP, *M. fascicularis*, male and female) between 7–16 yr of age, weighing 5–10 kg, and a control group of five ocular normotensive animals (ONT-NHP) were enrolled in this study. The animals are part of the Primate Colony from Nofovanny, Vietnam.

#### Animal preparation and restraint.

Experimentation on nonhuman primates (NHPs) was performed in accordance with the statement for the use of animals in ophthalmic and vision research approved by the Association for Research in Vision and Ophthalmology. The guidelines of the Animal Ethics Committee of the Singhealth Singapore Association for Assessment and Accreditation of Laboratory Animal Care were also satisfied (2015/SHS/1098; 2020/SHS/1580). OHT and ONT monkeys were trained and physically restrained in monkey chairs, necessary for measuring IOP and other ocular procedures. Before all ocular procedures, 1–2 drops of 1% xylocaine, as topical anesthesia, were applied to reduce possible discomfort to the animals during the procedure.

#### Intraocular pressure measurements and DcB instillation regimes.

IOP was measured using a calibrated Tonometer (Icare Tonovet). In all experiments, the mean IOP (±SE) was calculated from 6–8 consecutive IOP measurements at each time point.

Before commencement of the treatment, a once-daily baseline IOP measurement to both eyes of all animals was recorded consecutively for 3 days.

##### Acute setting.

One drop (30 μL) of the polysorbate vehicle or DcB dissolved in the vehicle was applied to both eyes. IOP was measured before instillation (Control) and at 2, 4, 8, and 24 h after instillation in both eyes. Two weeks were allowed for recovery between instillation at each concentration of DcB, namely, vehicle alone and 0.1, 0.2, 0.4, and 0.7 mM DcB at *weeks 1, 3, 5, 7, and 9*, respectively.

At 8 h after instillation, or at different times indicated in figure and table legends, slit-lamp examination was done to observe hyperemia, corneal edema and opacity, in vivo Pachymetry (CCT) to measure corneal thickness, analysis of aqueous flow rate (AFR), and recording of blinking frequency, pulse, and blood pressure.

##### Extended setting.

One drop (30 μL) of 0.02–0.2 mM DcB was applied once daily to both eyes for 7–10 days. IOP was measured before instillation (Control) and at 8 and 24 h after instillation in both eyes. At 8 h after instillation, or at different times indicated in figure and table legends, all other ocular measurements were carried out as in the acute setting. After each treatment, a 2-wk recovery period was allowed, after which IOP and other ocular parameters returned to the pretreatment values.

#### Clinical examinations.

After topical applications of DcB or vehicle, visual inspection of all eyes including signs of irritation, opacity, haziness, inflammation, or infection on cornea and conjunctiva was carried out daily.

#### Slit-lamp microscopy and anterior segment imaging.

Slit-lamp microscopic examination of the exterior, anterior chamber, and posterior chamber of the eyes was performed before the treatment and once daily during the treatment period. Anterior segment photographs and optical coherence tomography (RTVue Optovue Inc, Fremont, CA) of the eyes were performed before treatment (baseline) and once daily during the treatment period. The monkeys were also monitored for any gross changes such as eye discharge, squinting, or abnormal behavior suggesting pain or severe discomfort. A graded scale from mild to severe was used to grade conjunctival hyperemia/erythema.

#### Pachymetry.

Pachymetry was measured using Optovue optical coherence tomography (RTVue Optovue Inc, Fremont, CA) to observe the entire corneal thickness before and after dosing. Measurements of the central corneal thickness were made at various time points after drug instillation as indicated in figure and table legends.

#### Aqueous humor flow rate analysis.

The aqueous flow rate was analyzed by ocular scanning fluorophotometry (Fluorotron Master, OcuMetrics Inc, Mountain View, California) on two NHPs. Baseline fluorophotometry was done twice on each animal 1 wk before experiments. These values were averaged to give one baseline flow rate (µL/min). Measurement was then performed once after each drug instillation. The Fluorotron was approved for monkey use and this version only differs slightly from the human version in external features that make it appropriate for positioning to animal eyes. Briefly, 2% fluorescein eye drops were applied topically to both eyes of the NHPs. After 4 h, the cornea and anterior chamber were in a steady-state condition and the concentration ratio between the two was, therefore, constant as the fluorescein concentrations declined. Fluorotron readings were taken before instillation of the 2% fluorescein drops (auto-fluorescence), immediately after instillation, and at times between 4–8 h after instillation of the 2% fluorescein drop. The aqueous humor turnover rate (AFR, µL/min) was calculated using the designated Fluorotron program.^2^

### New Zealand White Rabbits

Animal handling and assessment of drug effects in New Zealand white rabbits were done as described previously (IACUC permission No. 04270911-2) (7). IOP measurements were made with a Pneumotonometer (Model 30, Reichert technologies) either after raising IOP with 4-AP (1 drop 40 mg/mL) (7) or on basal IOP. For comparison of the efficacy of DcB with timolol, dorzolamide, and latanoprost, single drops (30 µL) of DcB solutions at the indicated concentrations or of the clinically used drugs were instilled into the right eye (RE) and one drop of the vehicle into the left eye (LE) as control. For comparison of DcB with Latanoprost, three groups of five rabbits were treated every 90 min with 4-amino pyridine (4-AP) (1 drop 40 mg/mL) alone (Control) or with the drugs. Two hours after the initial instillation of 4-AP, one drop of 0.005% Latanoprost (XalatanTM, Pfizer) or after 2.5 h, one drop of 1 mM DcB, was instilled, alone or together, and IOP was measured at intervals of 1 or 2 h up to 10 h. Mean IOP values (± SE) were calculated from the five animals at each time point. For assessment of the K_0.5_ of DcB for the effect in the absence (basal IOP) and presence of 4-AP (raised IOP), increasing concentrations of DcB were instilled up to 1 mM. Curves were fitted to the function IOP_0_/IOP_DcB_= K_0.5/_K_0.5_ + [DcB] +constant.

### Porcine Ocular Tissue Preparations

Enucleated porcine eyes were freshly collected from a local slaughterhouse, and essentially as described in our published method of dissection, an intact annulus ring of the ciliary body and iris was excised and mounted in the fluid flow chamber (27, 28).

#### Simultaneous transepithelial electrical and AH fluid flow measurements.

The tailor-made fluid flow chamber used in the experiment was identical to that used in our previous studies (28). The excised ciliary body preparation was mounted into the chamber with an aperture area of 0.78 cm^2^. One half chamber was connected to a graduated capillary tube while the other half chamber was connected to an open bath (reservoir) to facilitate the administration of drugs. The bathing solution was filled on both sides of the chamber and bubbled with 95% O_2_ and 5% CO_2_ throughout the experiment at 23°C. The HEPES-buffered bathing solution contained (in mM) NaCl 113.0, KCl 4.6, NaHCO_3_ 21.0, MgSO_4_ 0.6, d-glucose 7.5, reduced glutathione 1.0, Na_2_HPO_4_ 1.0, HEPES 10.0, and CaCl_2_ 1.4. The pH of the bathing solution was adjusted to 7.4 with an osmolarity adjusted to 295 mosmol/kgH_2_O with d-mannitol.

Transepithelial electrical parameters, such as spontaneous electrical potential difference (PD), short-circuit current (Isc), and transmural resistance (R), were monitored with a dual-voltage current-clamp unit (World Precision Instruments). In addition, spontaneous transepithelial fluid flow was determined by measuring the changes in fluid level in the capillary tube. Drug administration was achieved by adding the drug(s) to the open bath reservoir, as appropriate (28). To minimize pressure differences across the ciliary body preparation, the fluid level in the capillary tube was adjusted to <5 mmHg throughout the experiment. The changes in fluid level (i.e., volumetric changes) were recorded once every 15 min for 1–2 h. Subsequently, the values were converted to rates of AH fluid inflow across the ciliary body and expressed in µL/h/preparation.

#### AH outflow facility measurements in ex vivo porcine eyes.

Freshly enucleated porcine eyes were thoroughly rinsed with PBS before removing the connective tissues and muscles from the globe. As described in our previous study (26), perfusion of pig’s eye was done with a cannulated 30-gauge needle inserted into the anterior chamber. The needle was connected to a glass syringe filled with bathing solution. The perfusion was controlled by a computerized syringe pump (Harvard Apparatus) via a pressure transducer (Honeywell). Throughout the experiment, the porcine eyes were perfused continuously with the HEPES-buffered bathing solution used for transepithelial measurements. Here, we adopted a constant flow perfusion system instead of our previous method (26). In this system, outflow facility was determined by measuring the IOP (pressure) at sequential flow rates (from 4 to 10 µL/min in 1 µL step). Stable pressure was obtained for at least 10 min at each perfusion flow rate. The AH outflow facility was then derived from the reciprocal of the changes in pressures at different flow rates (29).

#### Tissue isolation, membrane preparations, immunoblots, Na,K-ATPase Activity.

TM cells were isolated from freshly enucleated porcine eyes and characterized following previous protocols (30–32). After removal of the extraocular muscle, whole globe was rinsed with and immersed in phosphate-buffered saline (PBS) containing 1% PSG (100 U/mL penicillin, 100 mg/mL streptomycin, and 0.29 mg/mL glutamine). The entire globe was bisected at the equator, and then the anterior segment was vertically cut from the cornea to the sclera using a razor blade. The vitreous, lens, iris, and ciliary body were gently removed under a dissection microscope. Strips of TM tissue were carefully lifted out by forceps. TM tissues collected from 4–6 eyes were pooled as one sample and placed on a 60-mm culture dish in the incubator for ∼30 min to allow the TM explant to attach to the plate. Dulbecco’s modified Eagle’s medium (DMEM, Low glucose; Invitrogen), containing 1% PSG and 10% fetal bovine serum (FBS) (Invitrogen), was then added to cover the strips of TM tissues in the culture incubator overnight. Subsequently, additional media was gently added, and the cells were incubated for ∼1 wk. Once the cells migrated out, the TM strips were removed, and the medium was replaced every 3 days until confluence. The cells were trypsinized into a six-well plate for future use.

To verify the TM cell phenotype, the cells were treated with dexamethasone for 1 wk. The expression of myocilin protein was compared with dexamethasone-treated and untreated cells by Western blot. TM cells upregulated for the expression of myocilin were used for the experiment.

Porcine NPE and PE cells were isolated from ciliary bodies freshly dissected from enucleated porcine eyes, as described in detail in previous papers (8, 33). Briefly, the tissue was incubated with trypsin to release NPE and PE cells and the cell suspension was centrifuged on layers of Histodenz (15% and 30% wt/vol). NPE and PE cells were collected in well-separated layers at the aqueous–15% and 15–30% interfaces, respectively. Corneal endothelium cells were prepared from dissected cornea as described in Ref. 34.

The separated NPE and PE, TM, and corneal endothelium cells were then used for the preparation of membranes, and the ouabain-inhibited Na,K-ATPase activity was determined, essentially as described previously (7, 8). Briefly, for assay of Na,K-ATPase activity, membranes were first incubated with 0.8 mg/mL alamecithin for 30 min at 20–22°C. Membranes were then incubated in triplicate at 37°C for 10 min, without or with 1 mM ouabain, in a medium containing 130 mM NaCl, 20 mM KCl, 3 mM MgCl_2_, 25 mM histidine, pH 7.4, 1 mM EGTA, 1 mM azide, and 0.5 mM ATP. P_i_ release was assayed using PiColorLock gold malachite green (Innova Biosciences) and the ouabain-inhibited activity was expressed in units of µmoles·min^−1^·mg protein^−1^. The average values ±SE were calculated from three separate experiments (see Supplemental Fig S3*D*).

SDS-PAGE was run with 30-µg membrane protein per lane. The blots were probed with α1, α2, and α3 isoform-specific antibodies and calibrated using purified human α1β1, α2β1, and α3β1 0.05–0.2 µg per lane, as described in Ref. 35. The following isoform-specific antibodies were used: α1-specific monoclonal antibody (Millipore Cat. No. 05–369, RRID:AB_309699), α2-specific polyclonal antibody (Millipore Cat. No. 07–674, RRID:AB_390164), and α3-specific monoclonal antibody (Santa Cruz Biotechnology Cat. No. Sc-376967, RRID:AB_3095497).

### Statistical Methods

IOP, CCT, or AFR ± SE values depicted in tables represent the average from at least three separate determinations. Significance of the indicated differences was calculated by the unpaired Students *t* test (*P* values). *P* values <0.05 were considered significant. Curve fitting was done with KaleidaGraph.

## RESULTS

### Effects of Topical DcB in Nonhuman Primates

A major objective of the current work has been to assess efficacy of DcB in lowering IOP in the ocular hypertensive nonhuman primate model (OHT-NHP), compared with the ocular normotensive counterparts, ONT-NHP. Table 1 summarizes data on intraocular pressure (IOP), corneal thickness (CCT), and aqueous humor flow rate (AFR) of the OHT-NHP versus ONT-NHP animals. Apart from the large difference in IOP, it is noticeable that the corneal thickness (CCT) is not different, while the aqueous flow rate (AFR) is significantly lower in the OHT-NHP than in ONT-NHP animals. By visual examination, the appearance and clinical features of OHT-NHP and ONT-NHP eyes are similar.

**Table 1.** Normal values of intraocular pressure, corneal width, and aqueous humor flow rate

Measurement	ONT-NHP	OHT-NHP
IOP, mmHg	12.7 ± 0.25	26.0 ± 0.2 (*P* < 0.0001)
CCT, micron	444 ± 16	456 ± 2.3
AFR, µL/min	2.94 ± 0.23	2.3 ± 0.07 (*P* = 0.012)

Five ocular normotensive (ONT-NHP) and seven hypertensive (OHT-NHP) nonhuman primates were used for the analysis. Data are presented as average values ± SE (*n* = 5 or 7), respectively. *P* values were calculated by unpaired Student’s *t* test. AFR, aqueous humor flow rate; CCT, central corneal thickness; IOP, intraocular pressure; OHT-NHP, ocular hypertensive nonhuman primate; OHT-NHP, ocular normotensive nonhuman primate.

**Figure 1.**
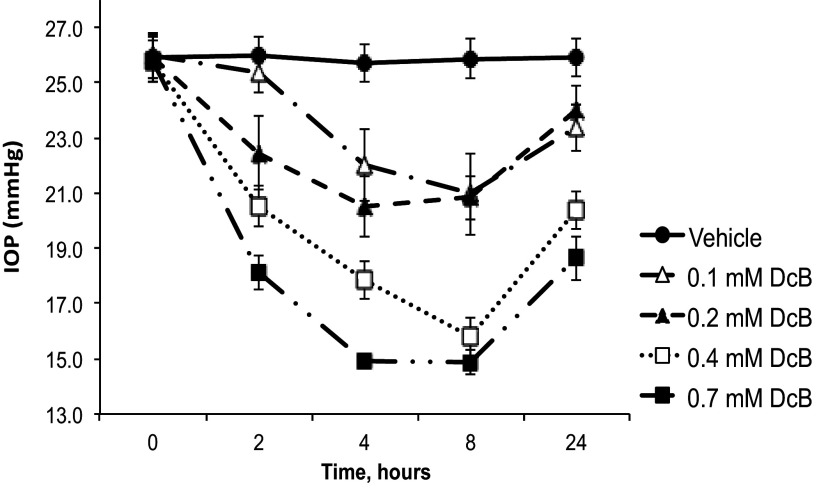
Acute effects of DcB on IOP in OHT-NHP. DcB at the indicated concentrations was dissolved in *vehicle A*. Using four OHT-NHP (8 eyes), IOP was measured at indicated times and CCT and AFR after 8 h. AFR, aqueous humor flow rate; CCT, central corneal thickness; DcB, cyclobutyl perhydro-1-4-oxazepine derivative of digoxin; IOP, intraocular pressure; OHT-NHP, ocular normotensive nonhuman primate.

Figure 1 and Table 2 present data on acute effects of increasing doses of DcB (0.1–0.7 mM) on IOP, CCT, and AFR in the OHT-NHP. A single drop of DcB was instilled into both eyes of four animals and the IOP was measured at the indicated times over 24 h. IOP was strongly reduced, in a dose-dependent fashion, reaching a minimum of ∼15 mmHg between 4–8 h after application of 0.7 mM DcB, a level close to that of the ONT-NHP, and then returned slowly toward the initial level. At 8 h after instillation, CCT and AFR were measured. The data summarized in Table 2 indicate a detectable increase in corneal thickness (CCT) and, in particular, an increased aqueous humor flow rate (AFR), to a value (2.89 µL/min) similar to that in the ONT-NHP.

**Table 2.** Acute effects of DcB on IOP, CCT, and AFR in OHT-NHP

DcB, mM	IOP, mmHg	CCT, microns	AFR, µl/min
0 (vehicle)	25.9 ± 0.67	455 ± 6.9	2.01 ± 0.23
0.1	22.0 ± 1.3	530 ± 17.9	2.28 ± 0.28
0.2	20.5 ± 1.0	559 ± 9.5	2.84 ± 0.28
0.4	17.8 ± 0.28	589 ± 16.6	2.88 ± 0.15
0.7	14.9 ± 0.63	574 ± 13.5	2.89 ± 0.25

DcB was dissolved in *vehicle A*. Data represents average values ± SE measured in four animals i.e., 8 eyes. Data on IOP are the values measured at 4 h after instillation, taken from Fig. 1. Values of CCT and AFR were obtained at 8 h after instillation. Data are presented as average values ± SE (*n* = 8). AFR, aqueous humor flow rate; CCT, central corneal thickness; DcB, cyclobutyl perhydro-1-4-oxazepine derivative of digoxin; IOP, intraocular pressure; OHT-NHP, ocular hypertensive nonhuman primate.

**Figure 2.**
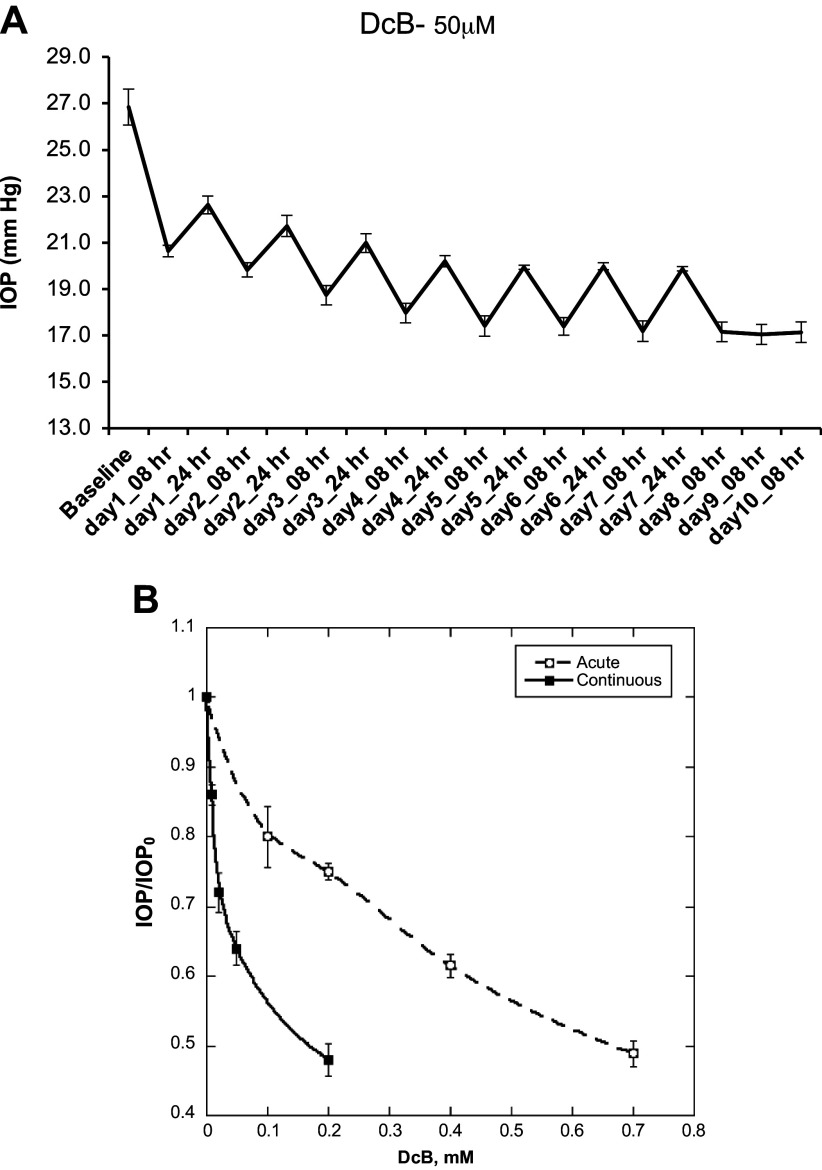
Effect of DcB on IOP of OHT-NHP during extended daily treatment. *A*: DcB (50 µM) dissolved in *vehicle B* was instilled daily for 10 days OHT-NHP (*n* = 4). IOP was measured 8 and 24 h after instillation. *B*: comparison of dose effects of DcB on IOP in extended versus acute settings. IOP data in the extended settings were obtained in four separate experiments (after 8 h on *days 4–7*). IOP data for the acute setting (at 4 h after instillation) was taken from Fig. 1. DcB, cyclobutyl perhydro-1-4-oxazepine derivative of digoxin; IOP, intraocular pressure; OHT-NHP, ocular hypertensive nonhuman primate.

Because reversal of IOP lowering in the acute setting of Fig. 1 is slow, and incomplete even at 24 h, daily instillation of DcB over a more prolonged period (e.g. 7–10 days) could be expected to lower IOP at a reduced dose. The experiments presented in Fig. 2 and data summarized in Table 3 tested and confirmed this assumption. For example, in the experiment in Fig. 2*A*, 50 µM DcB was applied once a day for 10 days and the IOP was measured at 8 and 24 h after instillation. IOP was strongly reduced, from 27.0 ± 0.77 to a minimum of 17.0 ± 0.44 mmHg after 4–5 days and remained at this steady level until the end of the experiment. As seen in Table 3, corneal thickness, CCT, measured 8 h after instillation over 7 days increased somewhat to a steady level of 542 ± 17.4 compared with the control at 456 ± 5.9 micron (16–19% in different experiments). Significantly, in parallel with reduction of IOP, the aqueous flow rate, AFR, was raised over 7 days from 2.30 ± 0.15 to 3.20 ± 0.17 µL/min (Table 3), a value similar to that in ONT animals (see Table 1). Similar experiments to that in Fig. 2*A* were done with different doses up to 200 µM DcB. The results in Fig. 2*B* shows the percent lowering of IOP in the steady-state (measured 8 h after instillation on *days 4 to 7*) using 8, 20, 50, and 200 µM DcB concentrations, respectively, compared with the value at 4 h in the acute experiment in the range 0.1–0.7 mM DcB taken from Fig. 1. Notably, the IOP was lowered to a constant level by ∼30% at 20 µM DcB, by ∼40% at 50 µM DcB and by over 50% at 200 µM, down to a value, 14–15 mmHg, near the IOP of ONT-NHP. By comparison, in the acute setting, the dose required to reduce IOP to the same level was much higher (0.7 mM) (Fig. 2*B*).

**Table 3.** Extended treatment of OHT-NHP with DcB and effect on IOP, CCT, and AFR

	IOP, mmHg	CCT, microns	AFR, µL/min
Control-OHT NHP	27.0 ± 0.77	456 ± 5.9	2.3 ± 0.15
Seven-day treatment with 50 µM DcB	17.0 ± 0.44 *P* < 0.0001 (*n* = 8)	542 ± 17.4 *P* = 0.0034 (*n* = 4)	3.2 ± 0.17 *P* = 0.0074 (*n* = 4)

**Figure 3.**
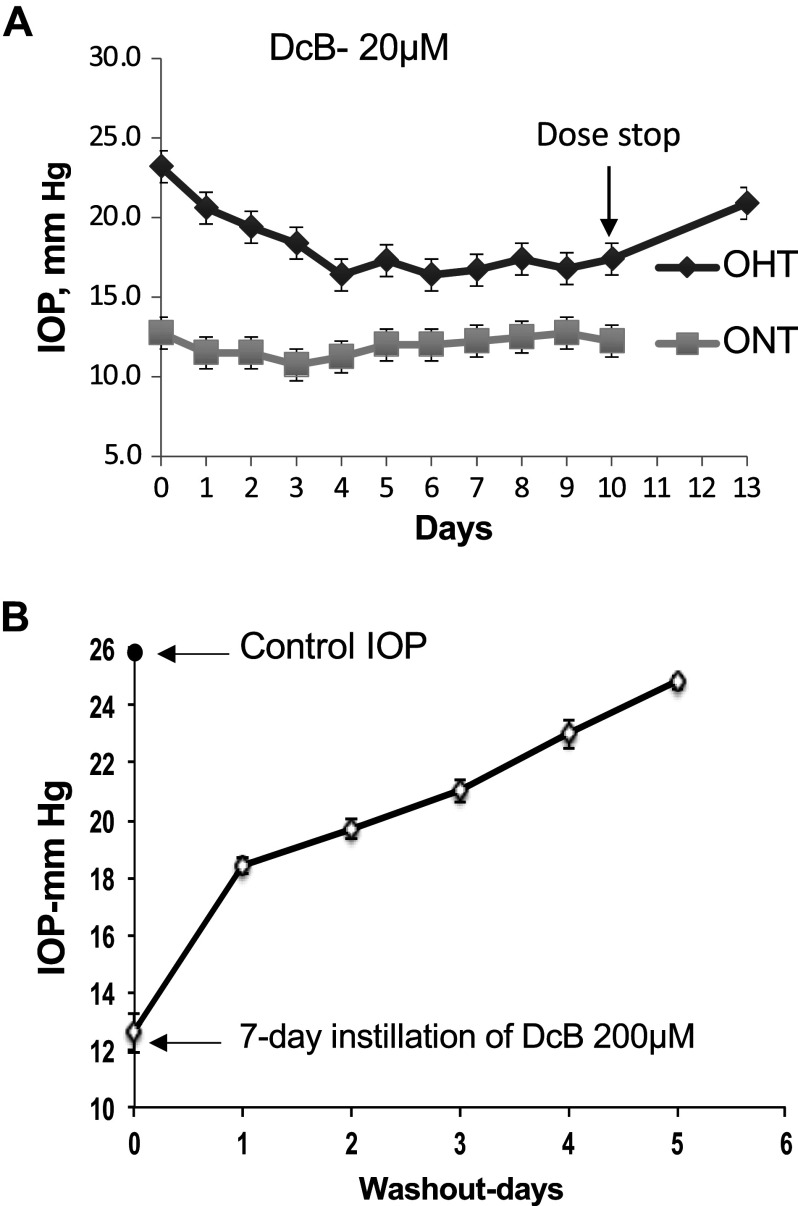
*A*: comparison of effect of DcB on OHT-NHP and ONT-NHP. DcB (20 µM) dissolved in *vehicle B* was instilled daily in both eyes of the OHT-NHP (*n* = 4) and ONT-NHP (*n* = 4) for 10 days. IOP was measured daily and, in the case of the OHT-NHP, also 3 days after cessation of instillation. *B*: rate of reversal of effect on OHT-NHP. DcB (200 µM) dissolved in *vehicle B* was instilled daily for 7 days into four OHT-NHP (8 eyes). After cessation of instillation, IOP was measured daily for 5 days. DcB, cyclobutyl perhydro-1-4-oxazepine derivative of digoxin; IOP, intraocular pressure; OHT-NHP, ocular hypertensive nonhuman primate; ONT-NHP, ocular normotensive nonhuman primate.

DcB was dissolved in *vehicle B* and instilled daily. IOP (see Fig. 3*A*), CCT, and AFR measurements were made at 8 h after instillation. The data represent the average steady-state values ± SE on *day 7* of instillation in four animals (i.e., 8 eyes). *P* values were calculated by unpaired Student’s *t* test. AFR, aqueous humor flow rate; CCT, central corneal thickness; DcB, cyclobutyl perhydro-1-4-oxazepine derivative of digoxin; IOP, intraocular pressure; OHT-NHP, ocular hypertensive nonhuman primate.

Two other points of interest arose out of the prolonged instillation experiments and are depicted in Fig. 3. In Fig. 3*A*, the effect on IOP in the OHT-NHP at a low concentration of DcB (20 µM), applied daily over 10 days, was compared with that in the ONT-NHP. Strikingly, while this dose reduced IOP in the OHT-NHP strongly (by ∼35%), there was little or no effect in the ONT-NHP. In the case of the OHT-NHP, the IOP was also measured 3 days after cessation of instillation, showing a slow but incomplete return to the initial level. Reversal of the IOP lowering effect was then examined more systematically in the experiment of Fig. 3*B*. DcB (200 µM) was applied daily for 7 days and, after cessation of dosing, reversal from the low steady level of IOP (c.13 mmHg in this case) was followed for the next 5 days. The experiment confirms that wash-out is slow with a half-time between 2–3 days. Note that this time course is not inconsistent with the slow reversal seen after cessation of treatment with 20 µM DcB, which is incomplete even after 3 days (Fig. 3*A*). These observations indicate that the wash-out time is not only slow but also largely independent of the DcB concentration.

**Figure 4.**
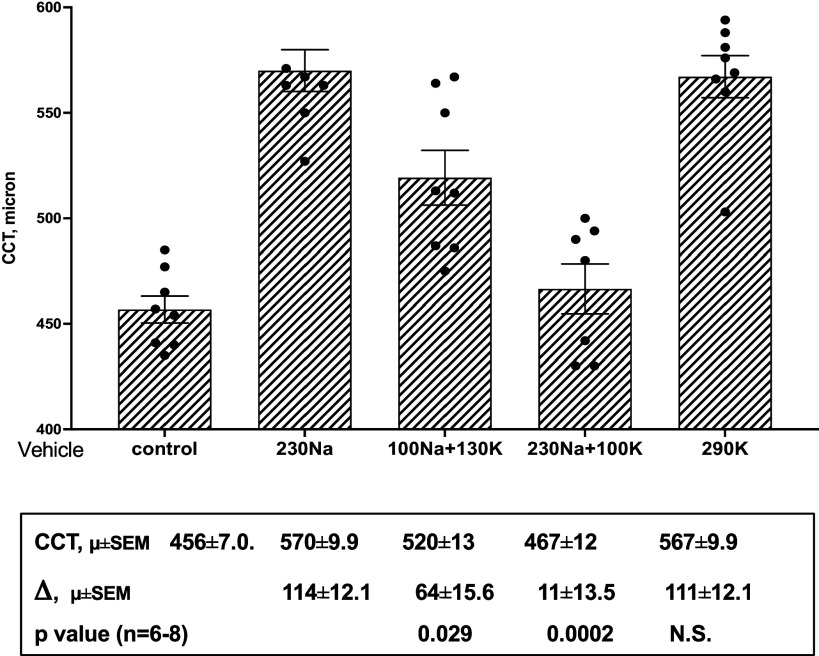
Effects of DcB dissolved in vehicles with different Na and K content on CCT of OHT-NHP. CCT was measured 24 h after instillation of DcB in four OHT-NHP (8 eyes). CCT values are averages ± SE in microns (µ) (*n* = 4), for DcB 200 µM dissolved in polysorbate vehicles containing 230 mM NaCl, 100 mM NaCl + 130 mM KCl, 230 mM NaCl +100 mM KCl, and 290 mM KCl, respectively. The Control CCT represents the average ± SE before instillation of DcB. *P* values for the differences in CCT between the vehicle containing 230 mM Na and the other vehicles were calculated by unpaired Student’s *t* test (*n* = 6–8), IOP was also measured 24 h after instillation of 200 µM DcB. CCT, central corneal thickness; DcB, cyclobutyl perhydro-1-4-oxazepine derivative of digoxin; OHT-NHP, ocular hypertensive nonhuman primate.

The increase in corneal thickness caused by the topical DcB (Tables 2 and 3), although mild, is an unwanted side effect and an effort to minimize it has been undertaken. Based on the well-known antagonism between cardiac glycosides and K ions (36), it was hypothesized that addition of KCl to the vehicle could protect the corneal endothelium Na,K-pump (37) locally against DcB, provided that the K ions penetrate the outer corneal epithelium. On the other hand, because K ions should be strongly diluted upon diffusion into the eye, the electrolyte composition of the vehicle would not be expected to interfere with binding of DcB to its target pumps or affect IOP lowering. This concept was explored in the experiments in Fig. 4, which depicts CCT values in microns (µ) 24 h after instillation of 200 µM DcB dissolved in vehicles containing 230 mM Na, 100 mM Na plus 130 mM K, 230 mM Na plus 100 mM K, or 290 mM K, respectively, compared with the Control, which depicts CCT prior to instillation of DcB. In preliminary experiments (not shown), the CCT was not affected by instillation of any of the vehicles themselves. As seen from the figure and CCT values, replacing part of the NaCl with KCl (vehicle with 100 mM NaCl plus 130 mM KCl) significantly reduced the DcB-induced increase in CCT (*P* = 0.029), while addition of KCl without reducing the NaCl (vehicle with 230 mM NaCl plus 100 mM KCl) was even more effective (*P* = 0.002), largely eliminating the DcB-induced increase in CCT. By contrast, replacing all of the NaCl with KCl (vehicle with 290 mM KCl) was not beneficial. The conclusion is that a vehicle containing both Na and K ions is optimal. As expected, the reduction of IOP was not significantly affected by the different vehicles falling from 26 ± 0.2 mmHg to between 18.3–19.5 ± 0.39 mmHg 24 h after instillation of 200 µM DcB (not shown).

Supplemental Figure S2 presents representative photographs of eyes of an OHT-NHP before and after daily instillation of 50 µM DcB for 6 days. Routinely, all monkeys treated with DcB were physically examined daily, pre-, and posttreatment, for any gross changes such as eye discharge, squinting, or abnormal behavior, and also clinical vital sign parameters such as body temperature, blood pressure, pulse (heart rate), and breathing rate (respiratory rate), behavioral changes, and systemic reactions. Slit-lamp biomicroscopic examination of the exterior, anterior chamber, and posterior chamber of the eyes was performed before the treatment and once daily during the treatment period to detect any signs of hyperemia/erythema, corneal opacities, inflammation, and change in conjunctiva and, also, for any gross changes such as eye discharge, squinting, or abnormal behavior suggesting pain or severe discomfort. The overall finding was that, for the 50 µM repeat dose-treated group, there was no significant change in any of the examined features, except for a mild increase in corneal thickness, consistent with the CCT measurements (Compare control and DcB treated in Supplemental Fig. S2 and Table 3). Moreover, there was no prominent systemic abnormality in any animal used in this repeat dose experiment, either during or after completion of the procedures described.

### Efficacy of DcB in Lowering IOP Compared with Common Glaucoma Drugs

**Figure 5.**
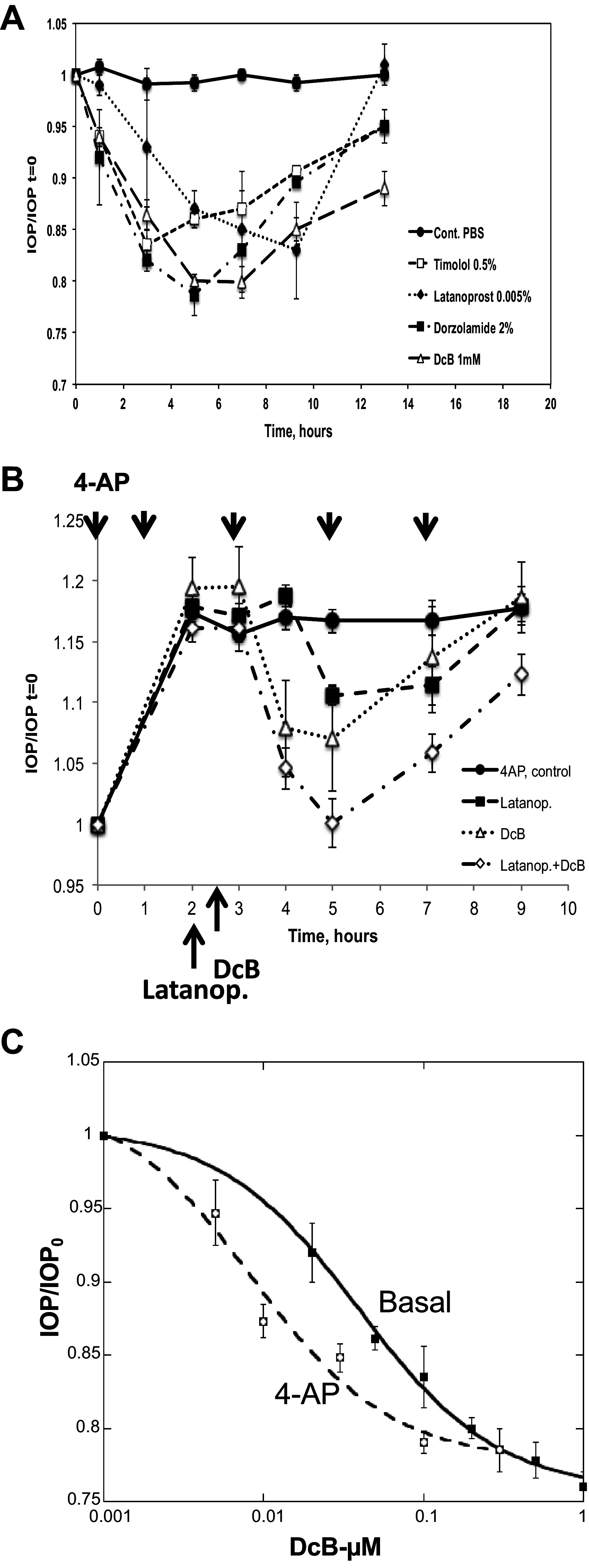
Comparison of effect of DcB and timolol, dorzolamide, and latanoprost on IOP in New Zealand Rabbits. *A*: 4-AP was instilled every 90 min for 13 h. IOP was measured in control animals every 2 h. In the test animals, 2 h after initiation of 4-AP instillation, DcB (1 mM) in *vehicle A*, or the other drugs were instilled and IOP was measured at the indicated times up to 13 h. Groups of five animals were used for each condition. *B*: 4-AP was instilled every 90 min for 9 h. Two hours after initiation of 4-AP instillation, latanoprost or DcB or latanoprost plus DcB was instilled and IOP was measured at the indicated times. Groups of five animals were used for each condition. *C*: DcB dissolved in *vehicle B* at indicated concentrations was instilled in rabbits treated with 4-AP (4-AP-induced ocular hypertension) or not (basal IOP), and IOP was measured after 4 h. K_0.5_ DcB values were evaluated using KaleidoGraph by fitting the data to the function IOP/IOPcontrol = [DcB]/([DcB]+ K_0.5_) + c. Groups of five animals were used for each concentration of DcB. DcB, cyclobutyl perhydro-1-4-oxazepine derivative of digoxin; IOP, intraocular pressure; 4-AP, 4-aminopyridine.

Observations on effects of DcB and other commonly used IOP-lowering drugs, latanoprost, timolol, and dorzolamide in the New Zealand rabbit model have allowed comparison of the IOP-lowering efficacy of DcB compared with the other drugs and also provided some mechanistic insight (Fig. 5). In basal conditions, the extent and duration of the effect of DcB were equal or greater than for latanoprost, timolol, and dorzolamide (Fig. 5*A*). In separate DcB dose escalation experiments, IOP was reduced from c.15 mmHg to 11–12 mmHg (i.e., by 20–25%) with a calculated K_0.5_ 39.8 + 4.3 µM DcB (see Fig. 5*C*). We have shown previously that the combination of DcB and latanoprost extends the duration of the effect compared with either drug alone (8). Nevertheless, reduction of basal IOP below a floor level 10–11 mmHg is difficult (8), making comparison of the extent of the effects hard to assess. Thus, it was of interest to compare the effects of DcB and latanoprost, alone or combined, in conditions of raised IOP. In rabbits, IOP can be raised pharmacologically, by pretreatment with 4-aminopyridine (4-AP), a compound that increases IOP by increasing AH inflow via the ciliary body (7, 17). For the experiment of Fig. 5*B*, 4AP was applied every 90 min leading to an increase in IOP from c.15 to 18 mmHg, maintained over the duration of the experiment (10 h). DcB 1 mM instilled after 3 h partially reversed the 4AP-induced increase in IOP over 4–5 h that then reverted to the 4-AP-induced control IOP after 9 h. The K_0.5_ DcB (8.4 ± 3.0 µM) measured in separate dose escalation studies was significantly lower than in basal condition (Fig. 5*C*). Latanoprost instilled alone also partially reduced IOP, but the combination of DcB and latanoprost was more effective, reducing the IOP to the basal level seen without 4AP, followed by the slower reversion to the 4-AP-induced control IOP level. A mechanistic implication of the additive effect of latanoprost and DcB on both the extent and duration of IOP reduction is that the two drugs act at different points of the aqueous humor flow cycle [namely, on the uveo-scleral (latanoprost) and trabecular meshwork (DcB) outflow pathways, respectively, see the discussion].

### Mechanism of Action of DcB

The DcB-induced increase in AFR and normalization of the AFR to the value characteristic of ONT-NHP (Tables 2 and 3) is suggestive of an increase in AH outflow facility rather than a decrease of the AH inflow. Therefore, a more detailed analysis of mechanism was undertaken, using isolated porcine ciliary epithelium to assess directly effects of DcB on AH inflow and ex vivo porcine eyes to assess effects on AH outflow facility.

**Figure 6.**
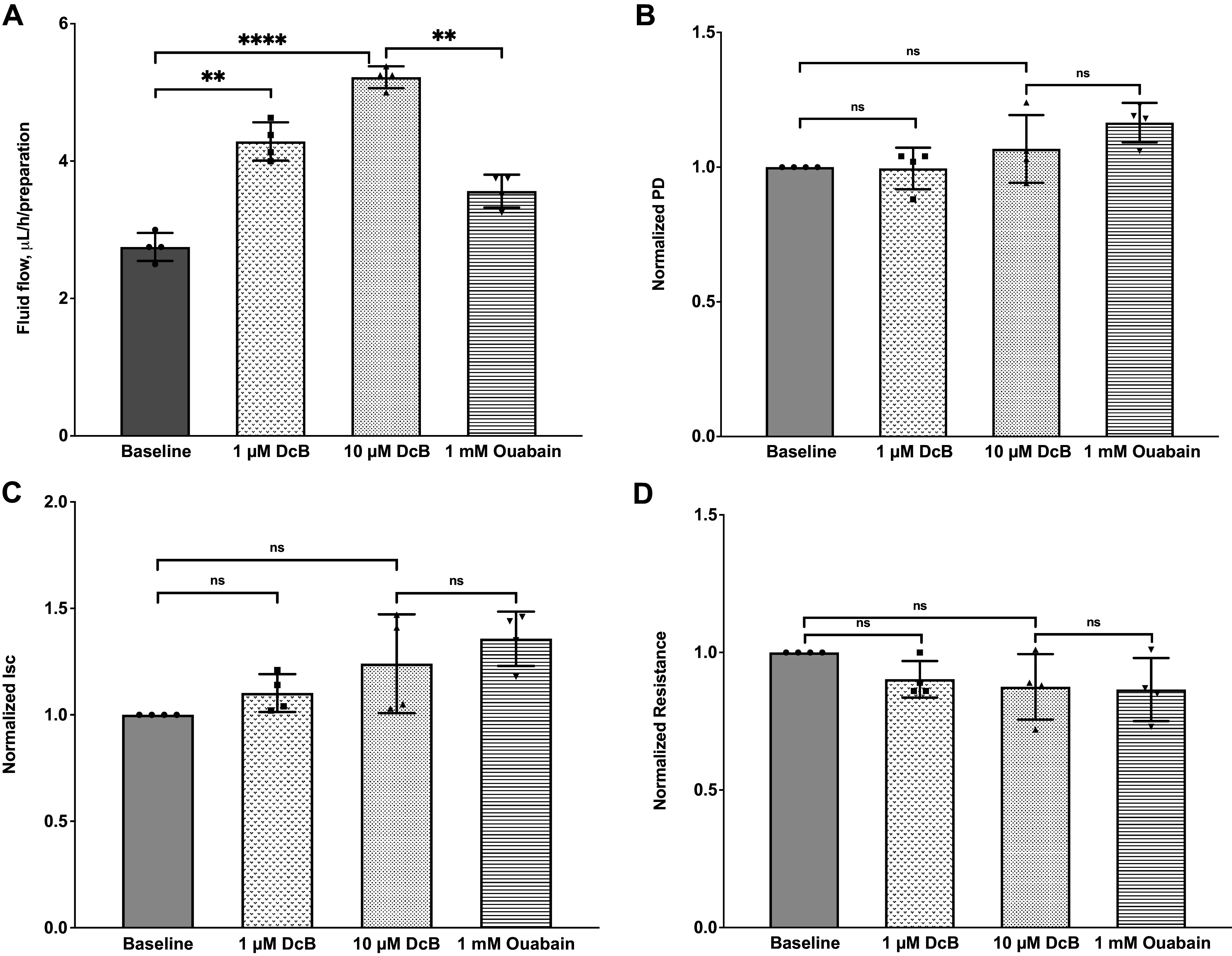
Effects of DcB and ouabain on transepithelial fluid flow and electrical parameters of porcine ciliary body. Effects of DcB on transepithelial fluid flow (*A*), potential difference (PD; *B*), short-circuit current (I_sc_; *C*), and transmural resistance (R; *D*). Fluid flow, PD, I_sc_, and R were measured simultaneously. Data were expressed in means ± SE (*n* = 4, **P < 0.01, ***P < 0.001, repeated-measures one-way ANOVA). DcB, cyclobutyl perhydro-1-4-oxazepine derivative of digoxin.

To assess whether DcB affects AH secretion, we have measured transepithelial AH fluid flow and electrical parameters in the excised porcine ciliary body/epithelium (38). As shown in Fig. 6*A*, a basal transepithelial fluid secretion of 2.75 ± 0.10 µL/h/preparation (from the stromal side to the aqueous side) was observed, consistent with our previous observations (25, 28). Note that the rate of fluid transfer is much lower than the AFR measured in vivo (2–3 µL/min). The difference may be attributable to incomplete exposure of the ciliary body in the fluid flow chamber, lack of a blood supply, or lack of hormones and other substances that stimulate active ionic movement (27, 38). Also, some AH may enter the anterior chamber passively through the surface of the iris without contributing to the AH flow in vivo via the ciliary body (39, 40). Nevertheless, this preparation has been found very useful for testing the effects of drugs, hormones, and inhibitors on fluid flow (25, 28).

The addition of DcB (1 and 10 µM) to the aqueous compartment, in contact with the NPE cell layer, triggered a significant increase in fluid secretion (Fig. 6*A*). At 1 µM, the fluid flow was increased to 4.28 ± 0.13 μL/h/preparation. Sequential addition of 10 µM DcB further stimulated AH secretion to 5.22 ± 0.80 μL/h/preparation, amounting to a ∼90% increase in AH secretion over the baseline value. By contrast, subsequent application of 1 mM ouabain to the aqueous surface significantly suppressed DcB-induced fluid secretion by ∼70% but did not completely block fluid secretion. Concomitantly, DcB did not elicit statistically significant changes in PD, I_sc_, and R at either concentration (Fig. 6, *B*–*D*; see the discussion for a possible explanation).

**Figure 7.**
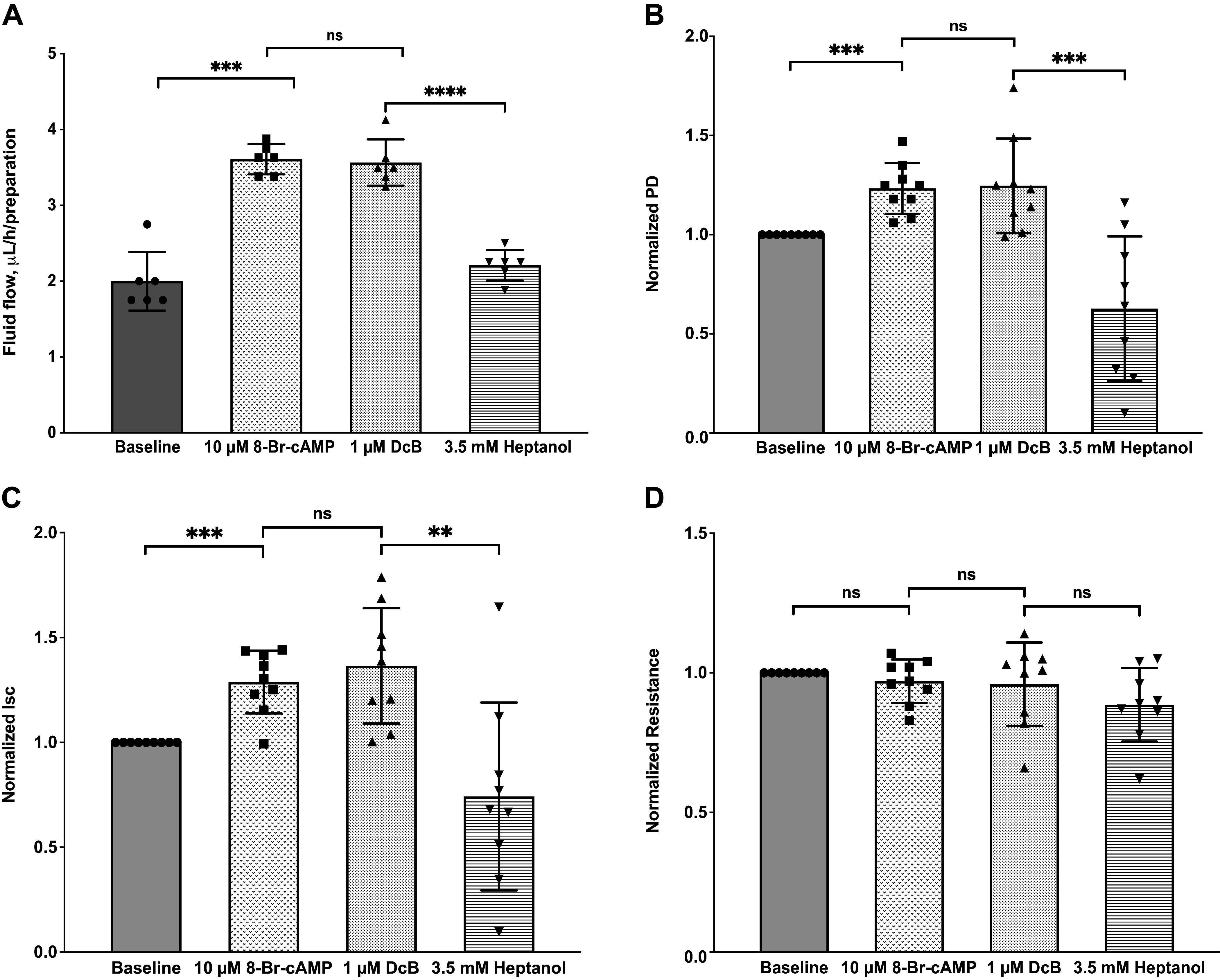
Effects of 8-Br-cAMP and DcB, when alone or combined, ouabain and heptanol on transepithelial fluid flow and electrical parameters of porcine ciliary body. Transepithelial fluid flow (*A*), potential difference (PD; *B*), short-circuit current (I_sc_; *C*), and transmural resistance (R; *D*). Fluid flow, PD, I_sc_, and R were monitored simultaneously. Data were presented in means ± SE (*n* = 6, **P < 0.01, ***P < 0.001, repeated-measures one-way ANOVA). DcB, cyclobutyl perhydro-1-4-oxazepine derivative of digoxin.

To test whether cAMP influences DcB-induced fluid secretion, we pretreated the preparation with 8-Br-cAMP (cAMP) (Fig. 7). As shown in Fig. 7*A*, the baseline fluid flow rate was 2.00 ± 0.16 µL/h/preparation. Addition of 10 µM 8-Br-cAMP to the aqueous surface significantly enhanced the secretion rate to 3.61 ± 0.08 μL/h/preparation, ∼80% higher than the baseline. Sequential addition of 1 µM DcB, however, did not produce any additive effect. Subsequently, addition of 3.5 mM heptanol, which is known to interrupt gap junctions between PE and NPE cells (41), totally inhibited the 8-Br-cAMP-stimulated fluid secretion, consistent with our previous findings of the transcellular 8-Br-cAMP-induced fluid secretion (28). In parallel with volumetric measurements, the changes in PD and I_sc_ after 8-Br-1cAMP and DcB treatment were consistent with the fluid flow secretion (Fig. 7, *B*–*D*).

**Figure 8.**
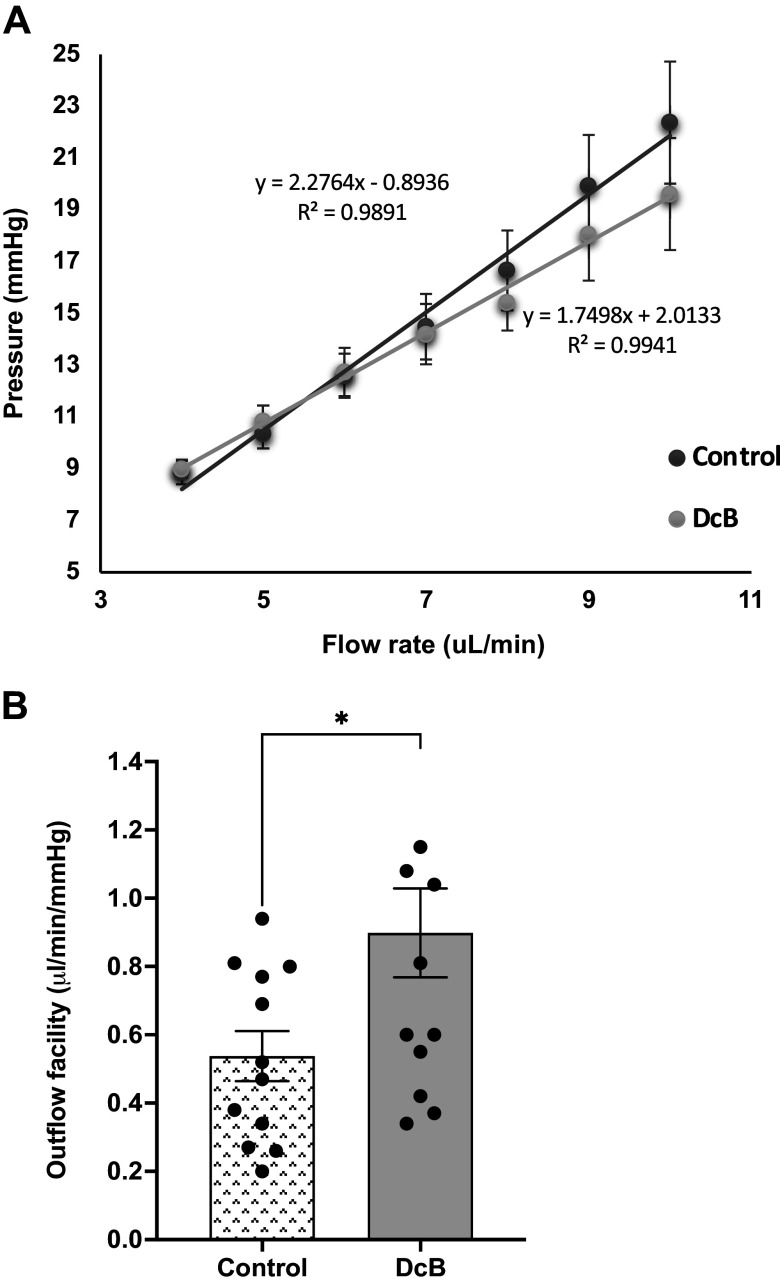
Effects of DcB on AH outflow facility in enucleated porcine eyes. *A*: the measured IOP (mmHg) at different flow rates (µL/min) applied in ex vivo porcine eyes in both control (*n* = 12) and DcB (1 µM, *n* = 13) conditions. Results were expressed in means ± SE. *B*: outflow facilities between control (*n* = 12) and DcB-treated (1 µM, *n* = 13) in ex vivo porcine eyes. Results were expressed as means ± SE (*P < 0.05, unpaired *t* test). AH, aqueous humor; DcB, cyclobutyl perhydro-1-4-oxazepine derivative of digoxin; IOP, intraocular pressure.

The changes in IOP (pressure) as a function of fluid flow rate measured in the isolated intact porcine eye and are summarized in Fig. 8. The slope of the best-fit line between pressure (mmHg) and flow rate used (µL/min) in DcB-treated eye (1 µM) was significantly lower than that of the control eye, indicating that DcB increases the outflow facility and leads to a reduced IOP. A good correlation with R^2^ > 0.98 was observed in both DcB-treated and control conditions (Fig. 8*A*). As shown in Fig. 8, the outflow facilities of the DcB-treated and control eyes were 0.90 ± 0.13 µL/min/mmHg (*n* = 13) and 0.54 ± 0.07 µL/min/mmHg (*n* = 12), respectively, showing that DcB triggers a 60–70% increase in outflow facility (Fig. 8*B*).

### Na,K-ATPase Isoform Content of Different Porcine Ocular Tissue Membranes

In relation to the mechanism of action of DcB, we have carried out semiquantitative Western blots of the α1, α2, and α3 content in membranes prepared from isolated porcine ciliary body NPE and PE cells, corneal endothelium, and TM membranes, using isoform-specific antibodies, and calibrated the band intensities with purified human isoform complexes (α1β1, α2β1, and α3β1) (Supplemental Fig. S3, *A–C*) (see Refs. 8, 35 for a similar approach). We have also determined Na,K-ATPase activities for all these membrane preparations (Supplemental Fig. S3*D*) As seen in Supplemental Fig. S3*C*, α1, α2, and α3 represent 17%, 64%, and 19% of the α subunit content in NPE, 100%, 0%, and 0% in PE, 44%, 0%, and 56% in corneal endothelium and 82%, 0%, and 18% in the TM membranes, respectively. Strikingly, α2 is detectable only in NPE membranes, where it represents the majority of the total α content with much lower levels of α1 and α3, whereas PE expresses only α1 and no α2 [similar to a previous finding in bovine NPE and PE cells (8)]. Corneal endothelium and TM membranes showed expression of both α1 and α3 but no α2. These expression profiles in the porcine ocular tissues are compatible with the more qualitative observations reported previously for different species, including bovine and rat ciliary epithelium cells (8, 13, 14), human corneal endothelium (15), as well as porcine, human, and bovine TM membranes, which express α1 and α3 (and β1 and β2) but no α2 (Dorette Z Ellis, unpublished, personal communication). The total α subunit content (α1+α2+α3) in the porcine membranes, NPE > corneal endothelium > TM>PE (Supplemental Fig. S3*C*), is also paralleled at least qualitatively by the order of Na,K-ATPase activities (Supplemental Fig. S3*D*).

## DISCUSSION

Elevated IOP has been consistently linked to both the development and progression of glaucoma and one noteworthy aspect of this association is the similarity in IOP levels between primates and humans (22). This feature, as well as their anatomical and physiological resemblance to humans, the occurrence of spontaneous OHT, extended lifespans facilitating long-term research, and the ability to conduct complex behavioral tests, underlines the relevance of NHP models and make them valuable tools for studying spontaneous ocular hypertension (OHT) and its similarity to human glaucoma, and potential glaucoma treatments (23).

### Effects of DcB in the OHT-NHP and ONP-NHP

The most striking feature of the effect of DcB on IOP in the OHT-NHP model is its large amplitude, over 40% in the acute setting at 0.7 mM DcB (Fig. 1 and Table 2) and over 50% in the 7–10-day continuous experiment at 0.2 mM DcB (see Fig. 2*B*, Table 3), and also slow reversal upon cessation of instillation (Fig. 3*B*). Furthermore, at a low concentration of DcB (20 µM) that gives a strong response in the OHT-NHP (c. 35%), the ONT-NHP animals show a weak effect if any, i.e., the latter are unresponsive or are much less sensitive to DcB (Fig. 3*A*). There are also differences between the different animal models in the magnitude of the response to topical DcB. For example, the maximal effect of DcB on IOP in the New Zealand rabbits, in either the basal state or with 4-AP-induced raised IOP (7, 8), is ∼20–25% of the control, namely, about half that in the OHT-NHP. In unpublished experiments, it has also been shown that DcB at 120 µM has little or no effect on IOP in Beagle dogs (quoted with permission by Santen Pharmaceutical Co., Ltd, Japan). These observations demonstrate that the choice of the specific animal model is an important consideration for testing drugs. In the case of DcB, the OHT-NHP is clearly the optimal animal model with an IOP response of greatest relevance to human glaucoma.

The experiments with the OHT-NHP model have also provided a strong hint at the possible mechanism of action of DcB. The finding that reduction of IOP to a level similar to that in ONT-NHP (Tables 2 and 3) is accompanied by an increase in the AFR from a level below that in ONT-NHP (Table 1) to a level similar to that in ONT-NHP (Tables 2 and 3) is essentially incompatible with the original hypothesis that lowering of IOP can be explained by a reduction of AH inflow (7, 8). Such a mechanism should be accompanied by a decrease in AFR. On the contrary, the observations on AFR imply that DcB increases AH outflow thus correcting the reduced AH outflow to that characteristic of the ONT-NHP (seen in Table 1).

The OHT-NHP model has been used previously to test a drug (24) but the current work appears to be the first example of a comparison of the effects of a drug on ocular hypertensive and normotensive animals. The different responsiveness to DcB in the OHT-NHP and ONT-NHP is clear cut (Fig. 3*A*) and may be significant pharmacologically, but the mechanistic basis of this difference is unclear. Note, however, that in rabbits, a significant difference in sensitivity to DcB is also observed for the basal versus the 4-AP-induced raised IOP, namely, K_0.5_ 39.8 ± 4.3 versus 8.4 ± 3.0 µM, respectively, Fig. 5*C*. As the mechanism of increased IOP in the OHT-NHP (decreased AH outflow) differs from that in 4-AP-treated rabbits (increased AH inflow), one could infer that there is a common factor, presumably the increased pressure itself, which sensitizes the TM to DcB and facilitates increased AH outflow.

The corneal side effect observed with the OHT-NHP is not seen in rabbits (8) or beagle dogs (Santen Pharmaceutical Co., Ltd., Japan, unpublished, quoted with permission) and may be species-specific. In any event, it is interesting that the inclusion of K with Na ions in the vehicle provided significant local protection against corneal thickening, without affecting the efficacy of DcB in reducing IOP. The K ions are presumed to penetrate the corneal epithelial layer and antagonize the binding of DcB to the Na,K-ATPase located on the inner corneal endothelial cell layer, which maintains volume equilibrium and visual clarity of the cornea, by the so-called endothelial pump mechanism (37). It is of note that local protection by K ions against a cardiac glycoside is not a new finding, as it was reported many years ago that K ions protect strongly against corneal swelling induced in rabbit eyes by topical ouabain (42).

### Mechanism of Action of DcB

We have adopted in vitro and ex vivo models to investigate the functional significance of DcB in porcine-excised ciliary epithelia and perfused eyes. Porcine eye is considered a good animal model for evaluating AH dynamics because it has similar anatomical characteristics to the human eye and shares many similarities in the composition of AH electrolytes (43, 44). Previously, we have demonstrated that currents stimulated by cAMP and forskolin in porcine ciliary epithelium are in good agreement with the results obtained in human ciliary epithelium (28, 45). The mechanism of action of 4-AP to raise IOP in rabbits, namely, stimulation of AH inflow associated with secretion of norepinephrine into the aqueous humor (17), suggests that in this animal model, raised cAMP in the NPE cells is also the cause of elevated AH inflow. Direct exposure of rabbit ciliary epithelium to β-adrenergic agonists is also known to raise cAMP levels (46). Finally, the glaucoma drug timolol is a β-adrenergic blocker, effective in both humans, rabbits (see for example Fig. 5*A*) and other species (5, 6) including cynomolgus monkeys (47), implying that the cAMP-stimulated AH fluid transport in ciliary epithelia is a feature common to humans and the different animal models used here.

Stimulation of AH inflow by DcB in isolated porcine ciliary epithelium (Fig. 6) fits well with the elevated AFR in DcB-treated OHT-NHP (Table 2), despite the difference in rates alluded to above. More importantly, although both of these sets of observations are mutually compatible, neither is compatible with the original hypothesis that DcB lowers IOP by reducing AH inflow (7, 8). On the contrary, they point to DcB-stimulated AH outflow facility as a likely alternative mechanism of action (see *Conclusions*).

Stimulation of AH inflow by DcB is itself somewhat surprising since active Na/K pumping powers transepithelial salt and water transport and full inhibition of Na,K-ATPase could be expected to impede AH inflow as it inhibits the transport of Na^+^ to the posterior chamber (48–50). However, it is also known that in basal conditions AH inflow is limited by the rate of chloride efflux at the aqueous surface of NPE cells (12). One can then propose the following hypothesis assuming that NPE Na,K-ATPase is only partially inhibited at 1–10 µM DcB in this system. Although partial inhibition of the pump may not itself significantly hinder AH inflow in ciliary epithelium, the treatment with 1–10 µM DcB could, plausibly, alter Na^+^ and concentrations of cellular electrolytes or other components in the NPE cells that further promote the release of Cl^−^ and AH inflow into the anterior chamber. A hint as to the factor(s) responsible for this effect is provided by the data in Fig. 7. The experiment shows that after exposure of the ciliary epithelium to 8-Br-cAMP, which significantly increases AH inflow (28), subsequent addition of 1 µM DcB has no further effect. Thus, the implication could be that DcB at 1–10 µM stimulates the production of cAMP in the NPE cells. Two mechanisms seem feasible. Inhibition of the Na,K-ATPase by cardiac glycosides saves ATP, the substrate of the adenylate cyclase. Alternatively, and perhaps more likely, adenylate cyclase is known to be Ca-dependent in various tissues (51), and in different tissues, such as the heart, inhibition of Na,K-ATPase by cardiac glycosides causes cellular Ca levels to rise due to increased Na/Ca exchange (on NCX), resulting from increased cytoplasmic Na concentrations (52).

The lack of significant effect of DcB on PD/Isc may imply that DcB stimulates AH secretion by simultaneously increasing both Na^+^ and Cl^−^ secretion, resulting in increased overall electroneutral salt transport and the associated fluid flow, but largely unchanged electrical parameters. In this connection, it is of interest that ouabain (5.10^−5^ M) was reported previously to stimulate transepithelial Na^+^ and Cl^−^ fluxes in rabbit’s iris-ciliary body (53, 54), suggesting that this phenomenon is not restricted to a particular cardiac glycoside (e.g., DcB) and is not specific to porcine tissues.

Another unexpected observation in Fig. 6 is that a high concentration of ouabain (1 mM) did not fully block and even slightly stimulated AH inflow, compared with the control, although much less than 1–10 µM DcB (Fig. 6*A*). One speculative explanation is based on the known anatomical differentiation of the ciliary body into plars plicata (folded) and plars plana (flat) segments. Thus, ouabain may not have full access to the pumps in the deep folds of the pars plicata NPE cell layer, even though these NPE cells contribute to the AH inflow (see, for example, Fig. 2D in Ref. 13). The net effect of the cardiac glycoside could then reflect stimulation by lower doses, and reduction by higher doses, down to a limit determined by access to the pumps within the structural infoldings of the ciliary body.

The direct observations on excised porcine eyes in Fig. 8 fit well with the inference that DcB lowers IOP by stimulating the AH outflow facility. The pressure-dependent effect seen in Fig. 8*A* shows that stimulation of AH outflow by DcB occurs via the conventional TM pressure-dependent path. Previously, in vitro experiments using perfused isolated porcine anterior eye segment and low-passage porcine TM cells showed that nontoxic concentrations of ouabain increase AH outflow facility, correlated with changes in the cytoskeleton and morphology of the TM cells (18). A subsequent report confirmed that both ouabain and a more reversible cardiac aglycone, strophanthidin, stimulated the AH outflow facility and suggested that the effects are not due to inhibition of the active cation transport, but rather to changes in TM cell scaffolding and/or signaling functions of the Na,K-ATPase (19).

It might seem paradoxical that a strongly α2-selective cardiac glycoside, DcB, acts in the TM segment cells that do not express α2 but only α1 and α3 (see Supplemental Figs. S3 and S4). Note, however, that selectivity for α2 is not absolute, and the following considerations suggest that the target of the IOP lowering effect of DcB is either or both α1 and α3. With simplifying assumptions on the percentage of instilled drug entering the eye and the volume of the anterior chamber, one can make a reasonable estimate of DcB concentrations to which the TM could be exposed. As an example, for a 30-µL drop of 200 µM DcB that suffices for a half maximal effect on IOP in the acute setting (Fig. 1) or a maximal effect in the extended setting (Fig. 2*B*), the estimated range is c. 400–2,000 nM.^3^ Isoform selectivity of DcB as judged by *K*_i_ values for inhibition of purified human isoform Na,K-ATPase activity is as follows (concentrations in nM ± SE, *n* = >3): α1β1, 135 ± 11; α2β1, 8.0 ± 1.3; α2β2 6.0 ± 1.0 and α2β3, 4.0 ± 0.15 (8, 9) and α3β1, 68.0 ± 9.0 (AK and SJDK, unpublished). The selectivity and *K*_i_ values for DcB of *M. fasciculata* and human isoforms can be assumed to be similar, especially as the protein sequences of *M. fasciculata* and human α1, α2, and α3 isoforms are virtually identical. Thus, at concentrations of 400–2,000 nM, both α1β1 and α3β1 in the TM segments in the OHT-NHP could be expected to be significantly bound with DcB and inhibited. With regard to the corneal swelling side effect, there is little doubt that inhibition of either α1 or α3 is responsible since corneal endothelial cells should be exposed to much higher concentrations of DcB than cells in the TM.

Overall, one can conclude that either or both α1β1 or α3β1 in the TM cells are the target of DcB and together with rapid corneal penetration, due to the high lipid solubility, these are critical factors responsible for the efficacy of DcB in lowering IOP. Consistent with the need for efficient corneal permeation, water-soluble cardiac glycosides such as 1 mM ouabain and even digoxin itself showed low efficacy for reducing basal IOP in vivo in the rabbits (7). On the other hand, experiments in rabbits with various perhydro-1,4-oxazepine derivatives of digoxin or digitoxin have not shown an absolute correlation between efficacy and predicted lipid solubility (clogP) (AK, and SJDK, unpublished), suggesting that additional factors may contribute to efficacy.

### Conclusions

One major conclusion of this paper is that in the OHT-NHP, and presumably other animal species, DcB reduces IOP by increasing the AH outflow facility via the pressure-dependent TM pathway rather than by inhibiting AH inflow, as hypothesized previously (7, 8). The detailed mechanism of action of DcB to both increase AH outflow and also stimulate AH inflow, namely, analysis of its effects on active ion pumping, cAMP-dependent and other signaling pathways, cell-cell interactions, cytoskeleton effect, etc., remain to be established and will be addressed in forthcoming studies.

DcB could become a viable candidate drug for the treatment of human glaucoma. It is significant that DcB lowers IOP potently via the main TM outflow pathway and, in this respect, differs from other currently used topical drugs with the exception of ROCK inhibitors (5, 6). Prostaglandin analogs such as latanoprost raise AH outflow facility via the uveoscleral route (5, 6), suggesting that combinations of DcB and latanoprost could be especially efficacious (inferred also from the data in Fig. 5*B*). Also, the slow reversal of DcB in the OHT-NHP (seen in Fig. 3*B*) implies that it could be instilled only once daily, ensuring optimal compliance.

Concerning possible toxicity, the corneal side effect observed in the OHT-NHP may be bypassed by the use of a slow-release formulation of DcB such as a cyclodextrin or lipid nanosphere complexes, which are currently being developed. Encapsulation of topical ocular drugs in slow-release formulations is a common technique used to reduce corneal irritation (55). Improved local corneal protection against DcB by K ions over time may also be achieved by adjusting the vehicle to an optimal K:Na ratio, or increasing K permeation with a selective ionophore, and combining both optimal local protection by K ions and a slow-release formulation of DcB. In relation to possible cardiac or general systemic toxicity, although cardiac glycosides such as digoxin used in humans to treat heart failure and cardiac arrhythmias must be carefully monitored to avoid cardiotoxicity, systemic toxicity is unlikely to be a problem after instillation into the eyes due to the large dilution in the systemic circulation. Indeed, Santen Pharmaceutical Co., Ltd. developed methods for assessing DcB concentrations in the general circulation and showed that the concentrations of DcB in the blood of cynomolgus monkeys after instillation into the eyes were very low, essentially negligible, (unpublished observation, quoted with permission). Thus, the concentration in the blood of humans after instillation in the eyes could be expected to be negligible.

With these caveats, DcB may become a promising candidate drug for use either as monotherapy or in combination with other drugs. Of course, in view of the species variation in mechanisms of AH formation, whether the response to DcB of humans with open-angle glaucoma is similar to that of the OHT-NHP model must be assessed independently.

## ETHICAL APPROVALS

Experiments using nonhuman primates at Singapore Eye Research Institute (SERI) were approved according to guidelines for Association for Assessment and Accreditation of Laboratory Animal Care (2015/SHS/1098; 2020/SHS/1580). Experiments using New Zealand rabbits at the Weizmann Institute were performed under authorization of IACUC (Permission No 04270911-2).

## DATA AVAILABILITY

Data generated for this study will be made available on reasonable request to the corresponding author.

## SUPPLEMENTAL DATA

10.34933/d2cbaedc-f02a-4be5-bb50-d1ef52f7dec0 Supplemental Figs. S1–S4: doi.org/10.34933/d2cbaedc-f02a-4be5-bb50-d1ef52f7dec0.

## GRANTS

This work was funded by research grants from the YEDA corporation, and a donation from Les.E and Cyndy Lederer, at the Weizmann Institute of Science (to S.J.D.K.); Health and Medical Research Fund (20212781), Region government (to C-W.D.); InnoHK initiative and the Hong Kong Special Administrative Region Government (to C-W.D.), 1-CD65 (to C-W.D.).

## DISCLOSURES

YEDA, the commercial arm of the Weizmann Institute, has filed two patents related to the work described in this paper. No conflicts of interest, financial or otherwise, are declared by the authors.

## AUTHOR CONTRIBUTIONS

V.A.B., C-W.D., and S.J.D.K. conceived and designed research; V.A.B., A.K., S.C., and D.M.T. performed experiments; C-W.D. analyzed data; A.K. interpreted results of experiments; A.K. prepared figures; S.J.D.K. drafted manuscript; V.A.B., C-W.D., and S.J.D.K. edited and revised the manuscript; V.A.B., H-L.L., D.M.T., A.L., C-W.D., and S.J.D.K. approved the final version of the manuscript.

## Footnotes

1 The mechanism of action of 4-AP has been shown to involve increased AH inflow, which results from the blocking of voltage-dependent K channels in sympathetic nerves of the iris-ciliary body, accompanied by increased norepinephrine release into the aqueous humor (17) and presumably, therefore, amplified cAMP production in the ciliary epithelium.

2 The abbreviation AFR refers throughout to the combined steady-state aqueous humor flow rate measured by the Fluorotron. AFR is dependent on rates of both AH inflow and AH outflow. AFR should be distinguished from the direct measurement of AH inflow across isolated ciliary epithelium and AH outflow facility measured in enucleated eyes.

3 Assume, typically, that 1–5% of the drug permeates into the eye and is diluted in the AH of the anterior chamber, with a volume of about 150 µL in cynomolgus monkeys. The estimated concentration range after instillation of 30 µL of 200 µM DcB is 400–2,000 nM. One can also take into account the wash-out time of the drug, which should be close to the AH turnover time. With an AFR of c. 2 µL/min, the half-time of AH wash-out is between 1–2 h. After 4 h, which is approximately two half-times, the predicted concentration of DcB would still be 100–500 nM.
